# Giant cell arteritis: pathogenic mechanisms and new potential therapeutic targets

**DOI:** 10.1186/s41927-017-0004-5

**Published:** 2017-11-28

**Authors:** Matthew J. Koster, Kenneth J. Warrington

**Affiliations:** Division of Rheumatology, Mayo Clinic College of Medicine and Science, 200 1st St SW, Rochester, MN 55905 USA

**Keywords:** Vasculitis, Giant cell arteritis, Pathogenesis, Therapeutics, Biologics

## Abstract

Giant cell arteritis (GCA) is the most common idiopathic systemic vasculitis in persons aged 50 years or greater. Treatment options for GCA, to-date, have been limited and have consisted primarily of glucocorticoids. Significant advances in the understanding of the genetic and cellular mechanisms in GCA are leading to identification of potential pathogenic targets. The recent success of interleukin-6 blockade in the treatment of GCA has opened the landscape to targeted biologic therapy. T cells, particularly T helper 1 and T helper 17 cell lineages have been identified as key inflammatory cells in both active and chronic vascular inflammatory lesions. Therapeutic agents, including abatacept and ustekinumab, which can impede both vasculitogenic cell lines are of particular interest. Inhibition of signalling pathways, including the janus kinase-signal tranducers and activation of transcription (JAK-STAT) and Notch pathways are evolving options. Tocilizumab has shown clear benefit in both newly diagnosed and relapsing patients with GCA and approval of this medication for treatment of GCA has led to rapid incorporation into treatment regimens. More information is required to understand the long-term outcomes of tocilizumab and other investigational targeted therapeutics in the treatment of GCA.

**Electronic supplementary material:**

The online version of this article (10.1186/s41927-017-0004-5) contains supplementary material, which is available to authorized users.

## Background

Giant cell arteritis (GCA) is the most common idiopathic systemic vasculitis in persons aged 50 years or older [1] and demonstrates a predilection for involvement of the aorta and its primary branches. The clinical presentation of GCA is variable and is based on the distribution of vascular involvement with most patients demonstrating classical cranial manifestations (headache, scalp tenderness, jaw claudication, vision loss) while others present primarily with constitutional and/or musculoskeletal symptoms. Less commonly, arterial occlusive disease of the extremities leads to symptoms of vascular claudication. Histopathologic examination of a temporal artery biopsy (TAB) specimen remains the gold-standard method of diagnosis, although vascular imaging studies are increasingly used in conjunction with, or instead of, an invasive biopsy. The aetiology and pathogenesis underscoring the development of GCA are still incompletely understood. Nevertheless, several advances in genetic and immunology research have led to a greater insight into the pathomechanisms that initiate and sustain vascular inflammation in GCA. Despite the availability of effective biologic agents for other autoimmune conditions, the treatment options for GCA, to-date, have been limited. Although glucocorticoids (GC) have been the standard-of-care for over six decades, treatment is not curative and is associated with significant morbidity. Therefore, the ongoing identification of key immune mediators in disease pathogenesis is encouraging the pursuit of clinical trials with targeted therapeutic agents. The aim of this article is to summarize the fundamental pathogenic mechanisms and new potential therapeutic options for the treatment of GCA. In order to accomplish this, we searched PubMed for the following search terms: “giant cell arteritis”, “large vessel vasculitis”, “temporal arteritis”, “arteritis” and “vasculitis”. Publications from the past 10 years were analyzed for pathogenic and therapeutic studies. Case reports were not included, unless providing unique insights into pathogenic pathways. The date of the last search was 1 July 2017.

## Main text

### Genetic associations

Several independent studies have implicated associations between GCA susceptibility and certain human leukocyte antigen (HLA) class I and class II alleles. In particular, carriage of HLA-DRB1*0401 and DRB1*0404 haplotypes have been consistently identified in multiple GCA cohorts [2–7]. A large-scale, international, genome-wide association study has further reinforced the importance of the HLA class II region [8]. Comparison of 2134 patients with GCA to 9125 controls demonstrated strong independent signals associated with conferring predisposition to GCA located in the HLA regions between HLA-DRA and HLA-DRB1 as well as between HLA-DQA1 and HLA-DQA2 [8]. Over-representation of these HLA class II genes strongly suggests that GCA is mediated by an antigen-driven immune response.

While HLA class II genetic factors have demonstrated the strongest association with risk of GCA development, several single nucleotide polymorphism risk signals in loci among non-HLA regions have also been recognized (Table 1). However, it should be noted that most studies evaluating genetic associations in GCA have included small groups of patients and will require validation in larger patient cohorts. Nevertheless, the wide array of potentially involved cellular pathways associated with GCA susceptibility underscores the polygenic nature and complex immunopathogenesis employing both the innate and adaptive immune response in this condition.

**Table 1:** Non-HLA genetic loci associated with giant cell arteritis

Non-HLA Locus	Function
Sample size ≥ 1000^a^	
Plasminogen (PLG) [8]	Lymphocyte recruitment, wound healing, fibrinolysis, angiogenesis
Prolyl 4-hydroxylase subunit alpha 2 (P4HA2) [8]	Collagen biosynthesis, folding of procollagen chains, hypoxia response
Tyrosine phosphatase non-receptor type 22 (PTPN22) [102]	Regulation of T and B cell receptor signaling
Interleukin-12B [8]	Th1 differentiation
Interleukin-17A ([103]; [104])	Th17 lymphocyte differentiation and maintenance
Interleukin-33 [105]	Th2 lymphocyte and mast cell activation, endothelial cell activation
Sample size ≥ 500	
NLR family pyrin domain containing 1 (NLRP1) [106]	Inflammasome assembly, activation of proinflammatory cytokines IL-1β, IL-18, IL-33
Sample size ≥ 250	
CC chemokine receptor 5 (CCR5) [107]	Proinflammatory chemokine activating cellular chemotaxis of macrophages, T lymphocytes, dendritic cells
Vascular endothelial growth factor (VEGF) ([108]; [109]; [110])	Neoangiogenesis, vascular remodeling
Interleukin-6 ([110]; [111]; [112])	Pleotropic pro-inflammatory cytokine
Sample size < 250	
Endothelial nitric oxide synthase (eNOS) ([113]; [114])	Synthesis of nitric oxide; regulation of endothelial cell vascular tone, cellular proliferation, platelet aggregation, and leukocyte adhesion
Tumor necrosis factor-a2 (TNFa2) [115]	Pleotropic pro-inflammatory cytokine
Interleukin-10 [116]	Regulation of Th1 and Th2 immunity, skews immune response to Th2 phenotype by inhibition of IL-12 production
Interleukin-18 [117]	Th1 differentiation

^a^If more than one study listed, then the sample size refers to the combined total of patients evaluated

### Infection

Isolation of identical T cell clones from different vasculitic sites suggests a response to a specific antigenic stimulus [9] and studies have proposed that arterial wall dendritic cells may be activated by environmental infectious agents or autoantigens [10]. Several microorganisms have been suggested as a possible infectious trigger including *Chlamydia pneumoniae* [11, 12], *Mycoplasma pneumoniae* [13], *Burkholderia pseudomallei* [14], parvovirus B19 [15, 16], herpes simplex virus [17] and Ebstein-Barr virus [18]. Although infection-induced autoimmunity leading to loss of self-tolerance through mechanisms of molecular mimicry, bystander T-cell activation and epitope spreading is plausible, direct evidence of such remains elusive. Indeed, attempts to identify pathologic organisms in temporal artery biopsy specimens have produced inconsistent results for any specific causal infectious agent [15, 19–21].

Varicella zoster virus (VZV) has received recent focus as a potential associated infectious aetiology. The presence of VZV antigen by immunohistochemistry was identified in 68 of 93 (73%) patients with histologically confirmed GCA and 45 of 70 (64%) patients with biopsy-negative GCA, compared to only 11 of 49 (22%) normal controls [22]. The same investigators identified VZV DNA by PCR amplification in a blinded analysis in 3 of 3 TAB-positive GCA patients and 4 of 6 TAB-negative GCA patients [23]. These investigators have proposed that the VZV is transported along the afferent nerves to the temporal artery inciting an inflammatory process resulting in arteritis. Consequently, Gilden et al. have advocated for use of the antiviral medication acyclovir in the treatment of patients with active or refractory GCA [24]. The presence of VZV as a causative agent for GCA, however, has not been substantiated by other groups. Muratore and colleagues evaluated 79 formalin-fixed and fresh-frozen temporal artery biopsies (34 TAB-positive GCA, 15 TAB-negative GCA, and 30 controls) by immunohistochemistry and PCR analysis [25]. Only 1 of 34 patients with TAB-positive GCA had evidence of VZV antigen whereas VZV antigen was not detected among any of the TAB-negative GCA patients or controls. Furthermore, VZV DNA was not found in any of the formalin-fixed or fresh-frozen TAB samples. In a recent prospective study, Procop and colleagues similarly did not identify VZV DNA from surgically sterile temporal artery and thoracic aortic samples from patients with large-vessel vasculitis [26].

In addition to histopathology evaluations, population level studies have failed to show a causal role of VZV in GCA. In comparing 204 cases of incident GCA diagnosed between 1950 and 2004 to 408 matched controls from the same geographic location, Schäfer and colleagues found no associated risk of incident VZV among patients with GCA compared to the general population [27]. Rhee et al. performed a population-based case-control study evaluating a larger sample of patients with GCA (*n* = 4559) and controls (*n* = 22,795) and similarly concluded there was minimal-to-no association of clinically overt VZV with GCA [28]. At current, conclusive evidence does not support direct infection with VZV as a causal process for the development of GCA and the use of acyclovir as an adjunct to, or in lieu of, immunosuppression is unsubstantiated and not recommended.

### Innate immune system

#### Vascular dendritic cells

Although the specific immunostimulatory trigger(s) is unknown, the immunopathology of GCA appears to originate from a dysregulated interaction between the vessel wall and both the innate and adaptive immune systems [29, 30]. Unlike small vessels which rely primarily on oxygen through luminal diffusion, large vessels require a microvascular network (vasa vasorum) to distribute oxygen to the media-adventia vascular cell layers. Arteries with vasa vasorum contain vascular dendritic cells (vasDCs) at the media-advential border where they are thought to participate in immune surveillance. In normal arteries, vasDCs are immature and lack the capacity to stimulate T cells [31] allowing arteries to maintain immune privilege and self-tolerance. In vasculitic lesions immune privilege is lost and vasDCs become activated via Toll-like receptors (TLRs), redistributing throughout the vessel wall [32]. Activated vasDCs are able to attract and activate T lymphocytes and macrophages through production of specific chemokine and cytokine signatures, providing a microenvironment necessary for initiating and sustaining arterial inflammation and granuloma formation [29]. This central role of vasDCs in arteritis has been demonstrated using a model in which human temporal arteries were engrafted into immunodeficient mice, following which targeted depletion of CD83+ vasDCs resulted in marked reduction of observed vasculitis. Due to their role early in the disease process, prevention of initial vasDCs activation is an unlikely therapeutic target. However therapies addressing persistently activated vasDCs remain a viable option.

#### Macrophages

GCA is a granulomatous vasculitis and multinucleated giant cells, a key feature of macrophage involvement, are a considered a pathognomonic hallmark of arterial lesions. Giant cell formation is present in approximately 50% of positive temporal artery biopsies. Macrophages recruited by activated vasDCs and T lymphocytes infiltrate the arterial wall through the vasa vasorum and further differentiate into M1 and M2 phenotypes according to the arterial microenvironment. In the adventitia, activated M1 macrophages primarily secrete proinflammatory cytokines (IL-1 and IL-6) [33] while M1 macrophages in the medial layer degrade the arterial matrix through secretion of matrix metalloproteinases and damage vascular smooth muscle cells (VSMCs) and endothelial cells (ECs) through local oxidative stress mechanisms [34]. On the contrary, M2 macrophages co-localize to the intima-media border and produce proangiogenic growth factors (vascular endothelial growth factor, fibroblast growth factor and platelet-derived growth factor) which results in myofibroblast proliferation, relocation, and the marked thickening of the arterial intima.

The emerging paradigm of personalized medicine is encouraging development of nanoparticle-based theranostic agents, which integrate therapeutic and imaging functionalities. These molecules are being explored in cancer and inflammatory diseases [35]. Selective targeting of specific macrophage phenotypes may overcome the toxicity associated with macrophage inhibition through non-selective cytotoxic drugs. Preclinical models for imaging and treatment of pathogenic macrophages have shown initial promise [36]; however, further advances in this evolving field will be needed prior to consideration of selective macrophage ablation, inhibition, or phenotype modulation in GCA.

### Adaptive immune system

#### T lymphocytes

Normal arteries are devoid of CD4+ T cell infiltration. In GCA, T cells are recruited via chemokines secreted by activated vasDCs, enter the arterial layer initially through the vasa vasorum, polarize into effector cell types and infiltrate the arterial layers. Two distinct CD4+ T effector cell subtypes have been identified as key regulators in vasculitic lesions of GCA; type 17 helper T cells (Th17) and type 1 helper T cells (Th1) [37]. These T cell lineages are likely to be stimulated by independent signals from distinct antigen presenting cells (APC) with Th17 cells dependent on IL-1β, IL-6, IL-21 and IL-23, while the Th1 pathway requires APCs secreting IL-12 and IL-18 [30, 37].

Studies conducted in patients with active, untreated GCA show that both Th1 and Th17 lineages are present in the inflammatory arterial infiltrates and are expanded in the peripheral circulation [38, 39]. The Th17 pathway appears to be very responsive to treatment and glucocorticoids (GCs) rapidly reduce the Th17 effector cytokine production of IL-1, IL-6, IL-17 and IL-23 [30, 38] with simultaneous depletion of both circulating and tissue infiltrative Th17 cells.

The rapid decline in these cytokines upon GC initiation contributes to the prompt decrease of systemic inflammatory features. Despite the effective reduction of the Th17 pathway, a Th1 cell response persists, both in blood samples and arterial specimens from patients treated with high-dose GC [38]. The Th1 cytokine signature identified in chronic vasculitis in GCA is associated with production of IL-2 and interferon-gamma (IFN- γ) and is poorly susceptible to GCs. It is likely that persistence of vascular Th1 cellular infiltrates despite prolonged courses of GCs is responsible for the chronicity and relapsing nature observed in GCA [30].

In addition to Th1/Th17 effector T cells, regulatory T cells (Tregs) appears to have an important role in GCA pathogenesis. Tregs, which are tasked with maintaining tolerance and prevention of autoimmunity, are decreased in the blood and arterial lesions of patients with GCA [39]. Compared to healthy controls, Tregs in patients with GCA appear to be defective and potentially pathogenic, exhibiting decreased proliferation and increased production of IL-17 [40]. While GC appear to be ineffective in modifying Treg dysfunction in GCA [39], IL-6 blockade was recently shown to revert the Treg abnormalities detected in active GCA [40].

Further confirmation that T cells are implicated in the development of large-vessel vasculitis is provided by the demonstration of T cell immune check point dysregulation in GCA. Checkpoint molecules, particularly cytotoxic T-lymphocyte-associated protein 4 (CTLA-4) and programmed death-1 (PD-1), play a key role in downregulating T cell activation, maintaining self-tolerance and limiting autoimmunity. T cell activation requires antigen recognition through the T-cell receptor as well as a second signal delivered by co-stimulatory molecules. CTLA-4 and its homologous protein, cluster of differentiation 28 (CD28), are expressed on activated T cells and participate in the co-stimulatory check point. Binding of CD28 to the CD80/86 molecules on APCs results in T cell activation and clonal expansion. On the contrary, down regulation of the immune response occurs through binding of CTLA4 to CD80/86, which results in T cell anergy. Checkpoint blockade immunotherapy is now being utilized in cancer treatments [41, 42]. Competitive inhibition of CTLA-4 by the immunostimulatory medication, ipilimumab, increases cytokine production, leads to persistent T cell activation and enhances T cell mediated immune responses to tumors [43]. However, use of ipilimumab has been associated with development of autoimmune conditions and organ-specific inflammation, including drug-induced giant cell arteritis [44].

The receptor molecule PD-1 provides inhibitory signals by binding to programmed cell death ligand 1 and 2 (PD-L1 and PD-L2), resulting in T cell anergy, apoptosis, or polarization to Tregs [45]. Recent transcriptome analysis of temporal arteries positive for GCA has demonstrated an inefficiency of the PD-1/PD-L1 checkpoint. Specifically, inflamed arteries of patients with GCA lack the inhibitory ligand PD-L1 and are enriched for PD-1–expressing T cells [46]. PD-1 blockade in a mouse model with engrafted temporal arteries worsened vascular inflammation, promoted PD-1+ effector T cells and increased effector T cell cytokines IL-17, IL-21, and IFN-γ [46]. These pre-clinical findings have been corroborated with recent reports of GCA developing in patients treated with pembrolizumab, a monoclonal antibody directed against PD-1 [47, 48].

#### B cells

Currently, the role of B cells in GCA is not fully understood. While plasma cells have been observed in positive temporal artery specimens in up to 83% of cases, the functional role of these cells remains indeterminate [49]. Associations with humoral immunity have been suggested by the presence of auto-antibodies directed against cardiolipin [50], ferritin [51, 52], endothelial cells [53] and recently 14–3-3 proteins [54]. However detection of auto-antibodies overall lacks specificity, has not been universally replicated, and is of unknown significance in the pathogenesis of GCA.

Although a major immunologic role of B cells is the production of antibodies, B cells can also regulate T cell responses in auto-immunity through the secretion of both pro-inflammatory (TNF-α and IL-6) and anti-inflammatory (IL-10) cytokines [55]. In a prospective study, patients with active GCA were observed to have low levels of circulating B cells, particularly IL-6 secreting effector B cells, with a return to near normal levels during GC-induced remission [55]. It is hypothesized that the B cells are distributed into secondary lymphoid organs and/or inflamed tissues and then on normalization of the inflammatory process return to the peripheral blood. However, the reservoir for B cells during active disease is incompletely known and remains an area of ongoing research.

One potential location for B cell redistribution during disease is within artery tertiary lymphoid organs (ATLO). Ciccia and colleagues evaluated temporal artery biopsies from fifty patients with active, untreated GCA and found distinct artery tertiary lymphoid organ structures in the medial layer among 60% of patients with biopsies positive for GCA [56]. These structures were organized into specific T cell areas, B cell follicles and associated with a network of follicular dendritic cells. Compared to patients without ATLOs and healthy controls, arteries with ATLOs had a high level of B cell activating factor (BAFF) and a proliferation inducing ligand (APRIL), proteins which are strongly correlated with the differentiation and proliferation of B cells. Production of these proteins was primarily driven by endothelial cells and vascular smooth muscle cells highlighting an interaction between myointimal cells and B cells, an area that requires further exploration and confirmation [56].

Although a potential role of B cells exists in the pathogenesis of GCA, the efficacy of B cell depletion is largely unknown. Rituximab, an anti-CD20 monoclonal antibody, has only been reported in two patients with GCA with observed benefit [57, 58]. Further investigation of this agent in prospective trials is needed before considering as a viable treatment option in GCA.

### Endothelial cells

Endothelial cells, the interface between the circulating blood and vessel wall, are active participants in the regulation of inflammation and angiogenesis. The intercellular adhesion molecules expressed on endothelial cells mediate leukocyte trafficking, migration, and development of inflammatory infiltrates. Increased expression of endothelin-1 and endothelin-B receptor immunoreactivity has been seen in higher concentration in the medial layer of temporal arteries from patients with GCA compared to controls and this immunoreactivity has been observed to correlate with the degree of systemic inflammation [59]. In addition, the endothelin system is increased at the protein level in temporal artery lesions in patients with GCA, creating a microvascular environment that is predisposed to develop ischemic complications, which is not immediately abrogated by glucocorticoid administration [60]. Indeed, upregulation of endothelin-1 in GCA lesions promotes migration of vascular smooth muscle cells to migrate towards the intima, leading to development of intimal hyperplasia and resulting in vascular occlusion [61]. Blockade of endothelin receptors using macitentan, a dual endothelin-A/endothelin-B receptor antagonist, has been shown to reduce vascular smooth muscle proliferation in biopsy-proven GCA and may prove to be a promising future adjunct in the treatment of GCA [62].

### Novel treatments

For over six decades, treatment with high-dose GCs has been the mainstay for both induction to remission and maintenance therapy in GCA. Unfortunately, GCs contribute substantial toxicity and GC-associated adverse events are nearly universal [63, 64]. In addition, despite adequate treatment with GCs, relapses are frequent [65] and chronic arterial inflammation can persist despite clinical and biochemical remission [38, 49]. Therefore, a great unmet need has existed for safe and effective GC-sparing agents for both induction and maintenance therapy in GCA.

Methotrexate was evaluated in 3 small clinical trials for treatment of GCA, and results were variable. According to expert consensus, methotrexate has been generally recommended as a second-line agent after GC in patients with relapsing or severely active disease or in patients with high-risk for GC-associated adverse events [66]. However, an individual patient data meta-analysis of the three clinical trials evaluating methotrexate found only a modest benefit in decreasing relapse risk and reducing GC exposure. Moreover, the effect of methotrexate on reducing relapse risk only became significant after about 48 weeks of treatment, and GC-related adverse effects were not diminished [67]. Limited to no benefit has been observed among other oral conventional immunosuppressive agents including azathioprine [68], leflunomide [69, 70] and mycophenolate mofetil [71].

**Fig. 1:**
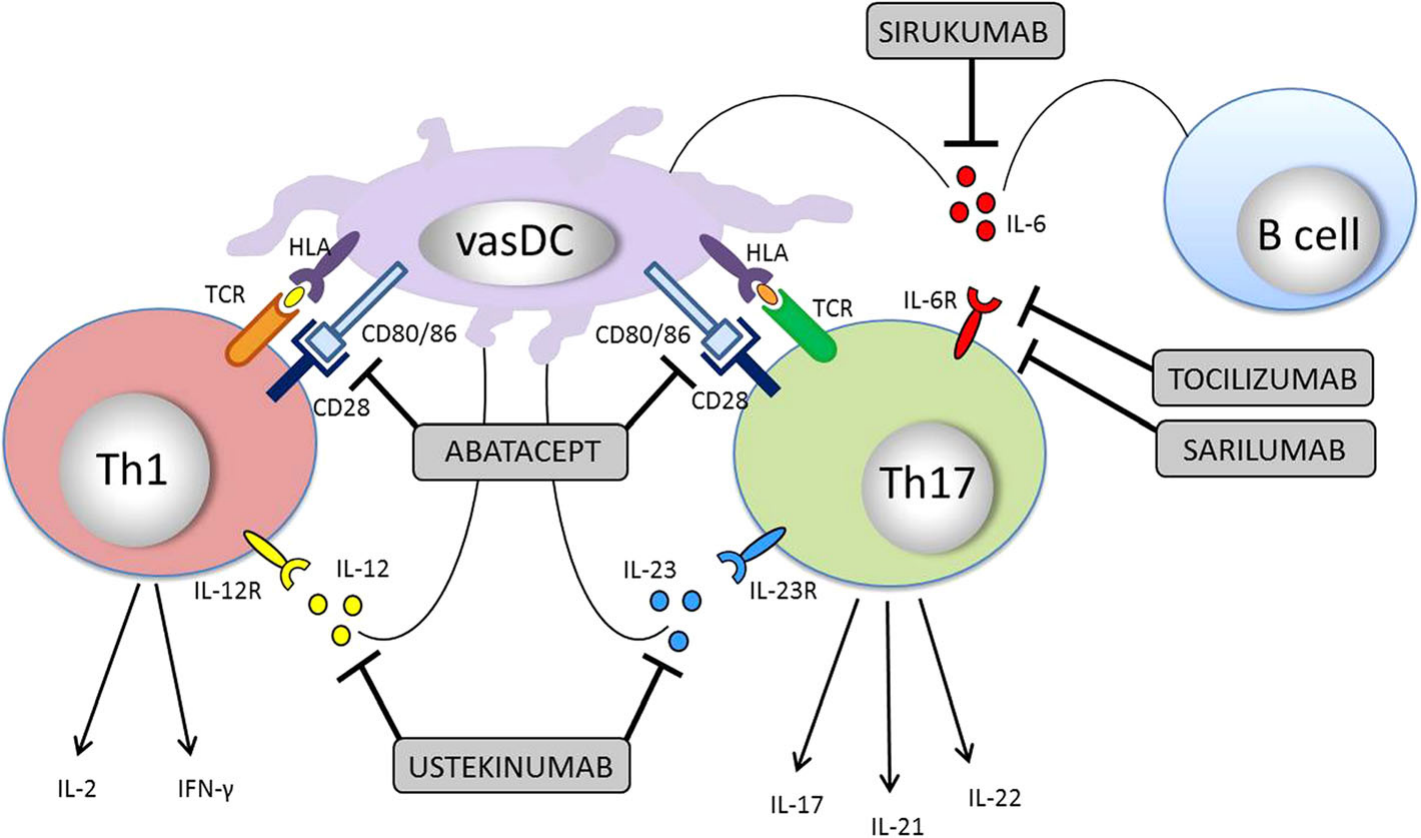
Vascular dendritic cells (vasDC) activate T helper 1 (Th1) and T helper 17 (Th17) cells by presenting antigen in the context of human leukocyte antigen (HLA) molecules. Abatacept can bind to CD80/86 and therefore block the required co-stimulatory signal required for T cell activation. Both B cells and vasDCs secrete interleukin (IL)-6 which stimulates Th17 cells via binding to its respective the T cell receptor (TCR). Sirukumab blocks soluble IL-6 whereas tocilizumab and sarilumab target the soluble and membrane-bound IL-6 receptor (IL-6R). IL-12 and IL-23 are secreted by vasDCs and antigen presenting cells and stimulate Th1 and Th17 cells, respectively. Ustekinumab binds the p40 subunits of both IL-12 and IL-23. Major effector cytokines produced by Th1 cells include IL-2 and interferon gamma (IFN-γ) and cytokines produced by Th17 cells include IL-17, IL-21 and IL-22

Due to the limited success of conventional immunosuppressive agents, targeted therapeutics have gained greater interest and investigation for control of GCA. Early reports of increased expression of tumor necrosis factor (TNF)-alpha in temporal artery specimens among patients with GCA [72] and dramatic benefit of TNF-inhibitors in other autoimmune diseases (e.g. rheumatoid arthritis) led to evaluation of TNF-alpha blockade in this condition. Unfortunately placebo-controlled trials utilizing TNF-inhibition with infliximab [73], etanercept [74], and adalimumab [75] have failed to show significant therapeutic efficacy in patients with GCA and did not result in significant reduction of GC use or adverse events. Despite the inadequate results of TNF-alpha inhibition, several other targeted therapeutics are being investigated (Fig. 1).

#### Interleukin-6 inhibitors

IL-6 is produced locally in the arterial vasculitic lesion [9], and is expressed in circulating monocytes from patients with GCA [33]. IL-6 is known to be a critical factor in the induction of acute-phase proteins, which are typically markedly elevated in patients with GCA and are used in clinical practice to monitor disease activity. Moreover, although IL-6 is highly elevated in untreated GCA, levels drop promptly with GC therapy. Indeed, IL-6 levels correlate with disease activity and can be a useful biomarker in evaluating disease activity [76, 77]. Tocilizumab (TCZ), a humanized anti IL-6 receptor antibody has shown efficacy in both newly diagnosed and relapsing patients in several small observational series [78–85] and two double-blind, placebo-controlled trials [86, 87].

In the first reported double-blind, placebo-controlled phase 2 trial evaluating TCZ in GCA, Villiger and colleagues randomized 30 patients with GCA (23 newly diagnosed, 7 relapsing) to receive prednisolone taper with intravenous tocilizumab (8 mg/kg/4 weeks) or prednisolone taper with placebo infusions [87]. Among the 20 patients receiving TCZ, complete remission at 12 weeks (85% vs. 40%) and relapse-free survival at 1 year (85% vs 20%) were significantly higher in the treatment group compared to placebo. On the contrary, serious adverse events were less frequent in the TCZ group (35% vs. 50%). Only one relapse was observed in the TCZ group and no relapses occurred after discontinuation of prednisolone in patients receiving TCZ.

The benefit and safety profile of TCZ has further been confirmed in the tocilizumab in GCA (GiACTA) trial [86]. GiACTA constitutes the largest international, multi-center, placebo-controlled trial in GCA to-date and the first to employ a blinded prednisone taper and a dual-assessor strategy to avoid bias created by knowledge of inflammatory markers [86]. In this phase 3 trial, Stone and colleagues recruited 251 patients (119 newly diagnosed, 132 relapsing) to one of four arms: weekly TCZ (162 mg, subcutaneous) plus a 26-week prednisone taper, every other week TCZ plus a 26-week prednisone taper, weekly placebo plus a 26-week prednisone taper, or weekly placebo plus a 52-week prednisone taper. Patients were diagnosed based on modifications to the 1990 American College of Rheumatology criteria for classification of GCA [88], which permitted inclusion of patients based on evidence of large-vessel vasculitis (LVV) on cross-sectional imaging. Whereas 62% of patients had a positive temporal biopsy, 37% of patients were ultimately enrolled on the basis of radiographic findings of LVV, regardless of temporal artery biopsy results.

The primary outcome, sustained remission at 52-weeks, was significantly higher among patients receiving weekly TCZ (56%) and every other week TCZ (53%) compared to the placebo groups (placebo plus 26-week prednisone taper, 14%; placebo plus 52-week prednisone taper, 18%; respectively). Disease flares occurred less frequently in patients receiving weekly TCZ (23%) and every other week TCZ (26%) compared to the 26-week (68%) and 52-week (49%) placebo arms. Over the 12-month trial, GC exposure in the treatment arms was approximately half compared to the placebo arms. Overall adverse events were frequent in all groups (92–98%); however, serious adverse events were lower among patients receiving weekly TCZ (15%) and every other week TCZ (14%) compared to placebo plus 26-week prednisone taper (22%) or 52-week prednisone taper (25%). Despite the accelerated GC taper, permanent vision loss did not occur in any arm and only one patient receiving every other week TCZ had transient visual loss, which recovered with glucocorticoids.

The landmark findings of TCZ safety and efficacy reported in GiACTA have led to the United States Food and Drug Administration to approve TCZ 162 mg administered subcutaneously weekly for the treatment of GCA; the first-ever medication to receive such distinction. The successful results of TCZ have prompted further investigation into additional novel anti-IL-6 molecules in this disease. A phase 3 trial utilizing sirukumab (human anti-IL-6 monoclonal antibody) is currently in progress (ClinicalTrials.gov Identifier NCT02531633) and an upcoming trial of sarilumab (anti-IL-6 receptor monoclonal antibody), is planned. The results of these studies will strongly influence whether additional candidate IL-6 blockers will be evaluated in GCA; including clazakizumab, olokizumab, and vobarilizumab. While the benefit of IL-6 blockade in treatment of GCA is apparent, 44–47% of patients receiving TCZ in GiACTA were still unable to reach sustained remission at 52 weeks [86]. In an earlier report, a patient with GCA who achieved apparent clinical remission on TCZ was noted to have evidence of persistent and active vascular inflammation at autopsy, despite normalization of inflammatory markers [85]. Active disease despite IL-6 blockade highlights that other immunologic pathomechanisms are present in ongoing inflammatory states in GCA and further investigation into other novel therapeutics is still needed.

#### Abatacept

Whereas the checkpoint inhibitor ipilimumab has led to the generation of arterial inflammation, inhibition of co-stimulatory T cell signalling via CTLA-4 is a possible target for controlling GCA. Abatacept is a fusion protein composed of the Fc region of the immunoglobulin IgG1 fused to the extracellular domain of CTLA-4 and blocks the co-stimulatory signal required for T cell activation. Langford and colleagues investigated the safety and efficacy of intravenous abatacept (10 mg/kg) in patients with newly diagnosed or relapsing GCA [89]. Following a remission induction phase, 41 patients in remission at week 12 were randomized to receive either placebo infusions or monthly abatacept. Relapse-free survival at 12 months, the primary outcome, was achieved by 48% on abatacept and 31% receiving placebo (*p* = 0.049). In addition, the duration of remission was on average 6 months longer in patients receiving abatacept compared to placebo. No difference in adverse events was observed. Although considered statistically and clinically significant findings, the effectiveness of abatacept in GCA needs to be confirmed in larger cohorts prior to incorporation of this agent in routine clinical care.

#### Ustekinumab

Tocilizumab, through blockade of IL-6R, mediates its effect largely on the Th17/Treg imbalance, without significant impact on the Th1 cellular pathway. On the contrary, ustekinumab, a monoclonal antibody directed against IL-12/23p40 has the potential to disrupt both Th1 (IL-12) and Th17 (IL-23) pathways. The effect of modulating the Th1/Th17/Treg imbalance has been demonstrated by Samson and colleagues in a refractory GCA patient treated with ustekinumab [90]. Compared to baseline, peripheral blood mononuclear cells evaluated after three injections of ustekinumab (45 mg weeks 0, 4, 16) demonstrated that Th1 and Th17 cells each fell by 50%, cytotoxic T lymphocytes reduced by 64%, and Tregs increased 5-fold. This dramatic improvement in T cell homeostasis is promising and the benefit of ustekinumab has been observed in a small open-label study. Conway and colleagues studied 12 patients with relapsing GCA and administered ustekinumab 90 mg at weeks 0, 4, and then every 12 weeks for a median of 8 months [91]. During the course of treatment GC requirements were noticeably reduced (median 23 mg prior, 5 mg after). Three patients were able to discontinue GC and eight patients were able to stop additional baseline immunosuppressives. No relapses were observed during treatment with ustekinumab but two of three patients experienced a flare following cessation of therapy. These findings demonstrate the potential efficacy of ustekinumab as a treatment option for GCA and a phase 2 trial to confirm these results is ongoing (ClinicalTrials.gov Identifier NCT02955147).

#### Janus kinase / signal transducers and activators of transcription inhibitors

The Janus kinase–signal transducers and activators of transcription (JAK-STAT) signalling pathway is involved in cellular regulation and has been implicated in the pathogenesis of several inflammatory and autoimmune conditions, including rheumatoid arthritis, inflammatory bowel disease, and psoriasis [92]. Ligand binding of immune relevant mediators (e.g. IL-2, IL-4, IL-6, IL-7, IL-9, IL-12, IL-15, IL-21, IL-23, IL-27, type 1 interferon, interferon-gamma) to their cell surface receptors leads to activation of associated JAKs [93]. The activated JAKs increase their kinase activity, recruit, bind and activate STATs. The STAT molecules then form hetero- or homo-dimers which translocate to the nucleus and induce transcription and expression of target genes, which in the context of pro-inflammatory cytokines can lead to increased inflammation and autoimmunity [94]. As a result, targeted intervention of the JAK-STAT signalling cascade is attractive and being investigated for several autoimmune and cancer disorders.

**Fig. 2:**
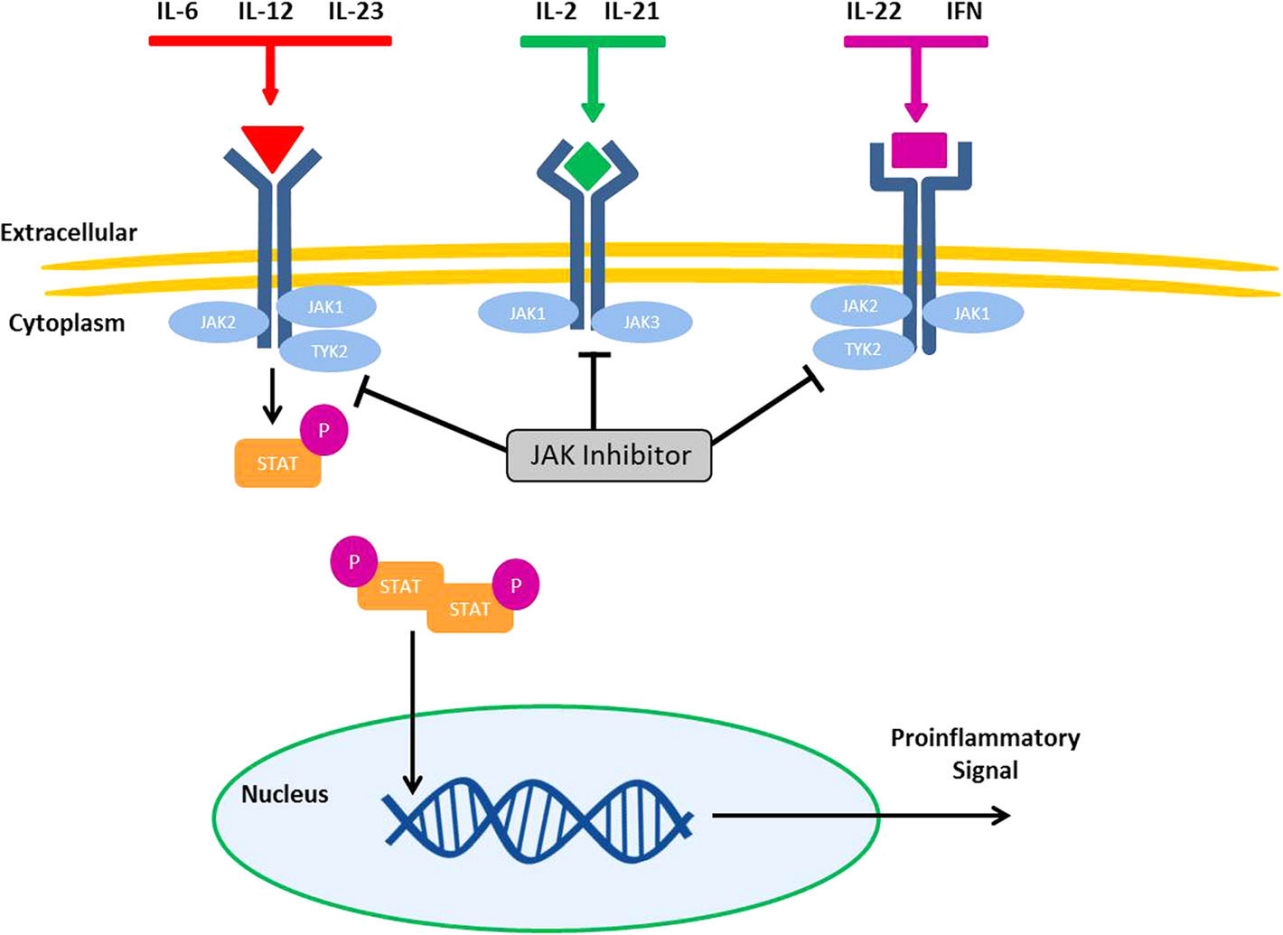
Effector cytokines can stimulate transcription of proinflammatory signals through the Janus kinase–signal transducers and activators of transcription (JAK-STAT) signalling pathway. Intracellular JAK and tyrosine kinase (TYK) proteins are activated when a ligand binds to its receptor which induces phosphorylation and activation, which in turn activate STAT proteins. The STAT proteins dimerize and translocate to the nucleus where they bind STAT-specific response elements in target gene promotors and regulate gene transcription; including proinflammatory signals. JAK inhibitors (e.g. baricitinib and tofacitinib) are able to abrogate signalling cascades from more than one effector cytokine pathway

JAK-STAT signalling has been identified as having a potential role in sustaining vascular inflammation. In particular, STAT-1 signalling appears to regulate the activity of vasDCs as well as controlling T cell trafficking and retention of inflammatory T cells in the vascular lesions [95]. Hartmann and colleagues evaluated the presence of STAT-1 transcripts in an experimentally induced vasculitis of human temporal arteries grafted in immunodeficient mice and found STAT-1 abundantly expressed in arteritic tissue lesions. Interferon-gamma, the major inducer of STAT-1, was, 10-fold higher in patients affected by GCA compared to controls. In the experimental model, while high-dose GCs effectively suppressed the activity of vasDCs and reduced IL-6 in the lesional tissue, the Th1 cellular infiltrate was not affected. Conversely, administration of the JAK/STAT inhibitor, tofacitinib, effectively prevented Th1 cell accumulation in the vessel wall and reduced interferon-gamma production [95]. Compared to TCZ, which mediates its effect primarily through the IL-6/gp130/STAT3 signalling axis [96]; JAK inhibitors can exert control over multiple pathogenic cellular lineages by impeding the proinflammatory signal of more than one cytokine signalling pathway (Fig. 2). The benefit of JAK inhibition in patients with GCA is currently unknown but a phase 2 trial evaluating baricitinib (oral selective JAK1, JAK2 inhibitor) in patients with relapsing GCA is currently ongoing (ClinicalTrials.gov Identifier NCT03026504).

#### Future treatment options

The Notch signalling pathway is critical for regulating cellular proliferation, differentiation, apoptosis, and homeostasis. Dysregulation of this highly preserved pathway has been associated with several malignancies and autoimmune conditions [97, 98]. Interaction between lymphocytes and vascular smooth muscles cells and endothelial cells involves the activity of the Notch pathway. Immunohistochemical and gene expression analysis of inflamed temporal arteries have identified overexpression of Notch receptors and their associated ligands (Jagged1, Delta1). Blockade of Notch signalling in a humanized vasculitis mouse model effectively depleted both Th1 and Th17 cells from vascular infiltrates [99]. In addition, it has been shown that systemic vascular endothelial growth factor (VEGF) can trigger aberrant Notch signalling through upregulation of the Notch ligand Jagged1 and induces pathogenic effector cell function via adventitial microvascular endothelial cells [100]. The arterial microvascular network appears to be essential in the recruitment of activated T cells and may therefore provide additional therapeutic targets. Multiple phase 1 and phase 2 trials evaluating different classes of Notch inhibition are underway in treatment of various forms of cancer, but Notch inhibition as of yet has not been attempted in patients with GCA.

The role of the pro-inflammatory cytokine IL-1-beta in GCA is uncertain; however, expression of IL-1-beta has been found in 60–80% of circulating monocytes in patients with untreated GCA and 20% of macrophages in temporal artery lesions produce this cytokine [33]. Limited information is available to determine the clinical efficacy of IL-1 blockade in patients with GCA but a small case series utilizing the IL-1 inhibitor, anakinra, showed benefit in three refractory cases [101]. A randomized, double-blind, placebo-controlled study evaluating gevokizumab (monoclonal antibody targeting IL-1 beta) for the treatment of patients with relapsing GCA is ongoing and has recruited 13 patients to date (EudraCT number 2013–002778-38).

## Conclusion

Recent advances have provided a greater understanding of the pathomechanisms involved in GCA; however, further studies are required to fully elucidate the aetiology and pathogenesis of this inflammatory vasculopathy. The complex interaction of genetics, vascular factors and immunologic pathways in this disease is responsible for the variability in both the clinical presentation and response to immunosuppressive therapy. After decades of treating GCA almost exclusively with glucocorticoids, clinicians are finally able to reach for novel and targeted biologic agents. IL-6 is a key mediator in GCA and tocilizumab is the first agent to show a profound effect on disease control and GC reduction. This agent is quickly being incorporated into the treatment algorithm of both newly diagnosed and relapsing patients with GCA. More information is needed to understand tocilizumab’s effect on long-term outcomes and the optimal length of therapy required for maintenance in this disease. Abatacept and ustekinumab have shown preliminary evidence of potential efficacy in the treatment of GCA but confirmation in larger trials is needed before either is utilized in clinical care. The proposed benefit of JAK-STAT inhibition seen in preclinical studies of vasculitis is encouraging and the evaluation of baricitinib in patient trials is underway. The era of glucocorticoid monotherapy for GCA may be coming to a close, a most welcome prospect for patients with this condition.

## Additional file

Additional file 1: Reviewer reports and AU response to reviewers. (DOCX 16 kb)

## Abbreviations

APC: antigen presenting cell
CTLA-4: cytotoxic T-lymphocyte associated protein-4
GC: glucocorticoids
GCA: giant cell arteritis
HLA: human leukocyte antigen
IL: interleukin
JAK-STAT: Janus kinase–signal transducers and activators of transcription
LVV: large vessel vasculitis
PD-1: programmed cell death-1
TAB: temporal artery biopsy
TCZ: tocilizumab
Th1: type 1 helper T cell
Th17: type 17 helper T cell
vasDC: vascular dendritic cell
VZV: varicella zoster virus
