# Supplementary material for: Giant cell arteritis: pathogenic mechanisms and new potential therapeutic targets
Source: BMC Rheumatol. 2017 Nov 28;1:2. doi: 10.1186/s41927-017-0004-5 (PMC6383596; doi:10.1186/s41927-017-0004-5)
Supplement: Supplementary file 1 — Reviewer reports and AU response to reviewers. (DOCX 16 kb) [file 41927_2017_4_MOESM1_ESM.docx]

**Open Peer Review reports**

Pre-publication versions of this article, reviewer reports and author comments to reviewers are available by contacting [info@biomedcentral.com](mailto:info@biomedcentral.com).

| Original submission |  |  |
| --- | --- | --- |
| 05 September 2017 | Submitted | Original manuscript |
| 19 September 2017 | Reviewed | Reviewer report - [Daniel Blockmans](javascript:popupReviewerInfo(446,81)) |
| 27 September 2017 | Reviewed | Reviewer report - [Sarah Mackie](javascript:popupReviewerInfo(324,81)) |
| 03 October 2017 | Author responded | Author comments - Matthew Koster |
| Resubmission – Version 2 |  |  |
| 03 October 2017 | Submitted | Manuscript version 2 |
| Publishing |  |  |
| 19 October 2017 | Editorially accepted |  |
| 28 November 2017 | Article published | 10.1186/s41927-017-0004-5 |

### How does Open Peer Review work?

Open peer review is a system where authors know who the reviewers are, and the reviewers know who the authors are. If the manuscript is accepted, the named reviewer reports are published alongside the article. Pre-publication versions of the article are available by contacting [info@biomedcentral.com](mailto:%20mailto:info@biomedcentral.com).

You can find further information about the peer review system at <https://bmcrheumatol.biomedcentral.com/about#peer-review+policy>
